# Protein Structure Prediction in Drug Discovery

**DOI:** 10.3390/biom13081258

**Published:** 2023-08-17

**Authors:** Alessandro Paiardini

**Affiliations:** Department of Biochemical Sciences “A. Rossi Fanelli”, Sapienza University, 00185 Rome, Italy; alessandro.paiardini@uniroma1.it

When the results of DeepMind’s AlphaFold2 at CASP were announced in 2020, the scientific world was so amazed by how effectively it performed that “it will change everything” became the motto for this revolution [1]. As a result, it should come as no surprise that “Protein Structure Prediction” was named Nature’s Method of the Year 2021.

Structure-based drug discovery (SBDD) is the one area of biology and medicine that is most expected to benefit and excel as a result of the developments of AlphaFold2 and comparable tools, such as RoseTTAFold [2]. However, since the accuracy of the residues’ conformations at the active sites remains a key limitation in SBDD, as does the inability to guess which conformational state of a protein these tools will predict, it is still necessary to associate and integrate previous physically based models and expert-driven knowledge with new machine-learning approaches, as well as experimentally derived structural data.

New approaches and tools, as well as developments in previously existing techniques for protein structure prediction and applications in immunology and virology therapeutic intervention targets, are described in this Special Issue.

In their work, Hey et al. conducted a study focused on Acute Respiratory Distress Syndrome (ARDS), a condition that often develops in severely ill individuals or those with significant injuries [3]. The researchers investigated the role of T-cells in the abnormal immune response leading to excessive tissue damage and the development of ARDS. The study specifically centered around Complementarity Determining Region 3 (CDR3) sequences derived from T-cells, which are crucial components of the adaptive immune response. Most of the diversity in T-cell receptors (TCRs) is found in the CDR3 regions of these receptors. To investigate the T-cell response in ARDS, the researchers used a novel technology called “immune sequencing” to analyze lung edema fluid samples. Their main objective was to explore the landscape of CDR3 clonal sequences present in these samples. They gathered more than 3615 CDR3 sequences from the studied samples. The results of the study revealed two main findings: the CDR3 sequences present in lung edema fluid showed distinct clonal populations, and these CDR3 sequences could be further characterized based on their biochemical features. Analyzing these CDR3 sequences provided insights into the T-cell repertoire driven by CDR3 in the context of ARDS. The study marked an initial step in utilizing this innovative technology to examine biological samples associated with ARDS, opening possibilities for future applications.

In their research, Naceri et al. focused on addressing the ongoing health threat posed by Influenza A viruses to both humans and animals [4]. They aimed to target the NS1 protein, a key viral component that suppresses the host immune response and facilitates viral replication. NS1 comprises a dimeric RNA-binding domain (RBD), which is structurally stable and conserved in sequence. Additionally, NS1 has two effector domains linked to the RBD by flexible linker regions, leading to variations in its structure and form. The researchers previously identified a potential drug-binding site within the RBD interface of NS1. This site could serve as a target to disrupt RNA binding and inhibit NS1’s functions. The primary objective of their study was to confirm the existence of this druggable site across various NS1 sequence variants to develop a universal therapeutic compound that remains effective despite sequence variations and structural flexibility. To achieve this goal, Naceri et al. employed a multifaceted approach. They utilized a set of four NS1 full-length structures and combined several bioinformatics methods. These methods included tracking binding pockets through molecular dynamics simulations, predicting and classifying druggability, and analyzing the results. Their comprehensive protocol successfully verified the presence of a large binding site that is highly druggable and consistently shared among different forms of NS1. This discovery holds promise for the development of a robust therapeutic strategy targeting NS1, which could potentially lead to a universal treatment that remains effective against various NS1 sequence variants and structural conformations.

Bhowmick et al. addressed the significant threat posed by the rapidly mutating SARS-CoV-2 Omicron variant, which has impacted societies worldwide [5]. They aimed to enhance our understanding of the virus and aid in therapeutic development by employing computational strategies to predict mutagenesis, focusing on the structural diversity of SARS-CoV-2 and its interactions with the human receptor. The researchers utilized protein structure prediction algorithms alongside molecular docking techniques to investigate the effects of mutations in the Receptor-Binding Domain (RBD) of SARS-CoV-2 and its interactions with the angiotensin-converting enzyme 2 (ACE-2) receptor. To achieve this, they generated RBD structures for different naturally occurring variants of SARS-CoV-2, starting from the WUHAN-Hu-1 reference, using the trRosetta algorithm. Using docking (HADDOCK) and binding analysis (PRODIGY), the study highlighted essential interactions within the Receptor-Binding Motif (RBM) between the predicted RBD sequences and the ACE-2 receptor. They proceeded to experimentally test the effects of mutagenesis at conserved residues in the Original, Delta, and Omicron variants, specifically introducing mutations P499S and T500R. The results demonstrated that these mutations led to stronger binding and interactions with the ACE-2 receptor. Furthermore, the researchers conducted in vitro tests on the T500R mutation to verify its binding and transmissibility in cells. The experimental outcomes aligned with the in silico analysis, providing confidence in the predictions. Their study showcased the utilization of the trRosetta algorithm to predict protein structure and future mutations at the RBM of SARS-CoV-2.

In their research, Dick et al. focused on the HIV-1 capsid (CA) protein as a potential therapeutic target [6]. The study aimed to create, produce, purify, and characterize CA proteins from various isolates representing clade A1, A2, B, C, and D of HIV-1. The researchers generated new CA constructs for these diverse HIV-1 subtypes, which could serve as valuable resources for future inhibitor design and investigations into the structural and biochemical properties of CA proteins across different genetic variants of the virus. They employed computational modeling to explore differences in CA assembly and the binding of specific inhibitors such as PF-74, CPSF-6, and NUP-153 among these various clades. A notable finding from their study was that HIV-1 CA from clade A1 did not bind to NUP-153. This observation suggested a potential alteration in the mechanism by which CA core structures are imported through the nuclear pore complex for viruses from this particular clade. In summary, Dick et al. demonstrated that models of the HIV-1 CA protein generated through computational methods for clades other than the commonly studied clade B can be valuable tools. These models aid in understanding and predicting biological processes, as well as in the design and mechanism of action of antiviral drugs targeting CA.

Finally, in their review, Rosignoli and Paiardini discussed the significant growth in the availability of structural bioinformatics tools and software over the past decades [7]. This proliferation has created a complex landscape of methods, algorithms, libraries, web resources, and pipelines, accessible to a wide range of life scientists. The authors highlighted the role of PyMOL, a widely utilized software for visualizing and analyzing biomolecules, in becoming a pivotal platform for translating expert knowledge into a user-friendly molecular graphics tool. The review focused on PyMOL’s increasing importance as an environment that supports various aspects of structural bioinformatics analyses. The authors specifically outlined the plugins and features that contribute to PyMOL’s suitability for these tasks. They highlighted how PyMOL’s capabilities make it a valuable resource for researchers engaged in exploring and understanding complex biomolecular structures.

The mentioned works have collectively made significant contributions to the field of Protein Structure Prediction in Drug Discovery. They have advanced our understanding of complex biological systems, paving the way for more effective therapeutic interventions. By exploring the structural diversity of viral proteins, such as the HIV-1 capsid and SARS-CoV-2 NS1 protein, these studies have uncovered potential drug targets and sites for intervention. Utilizing computational approaches, they have elucidated the intricate interactions between proteins and their binding partners, providing insights into mechanisms of action for inhibitors.

The studies also underscore the importance of utilizing advanced technologies, such as AlphaFold, PyMOL, and trRosetta algorithms, to elucidate intricate molecular structures and interactions. By bridging computational predictions with experimental results, they provide a comprehensive understanding of protein behavior and folding, which is instrumental in designing effective drugs.

## References

[1] Jumper J., Evans R., Pritzel A., Green T., Figurnov M., Ronneberger O., Tunyasuvunakool K., Bates R., Žídek A., Potapenko A. (2021). Highly Accurate Protein Structure Prediction with AlphaFold. Nature.

[2] Baek M., DiMaio F., Anishchenko I., Dauparas J., Ovchinnikov S., Lee G.R., Wang J., Cong Q., Kinch L.N., Schaeffer R.D. (2021). Accurate Prediction of Protein Structures and Interactions Using a Three-Track Neural Network. Science.

[3] Hey S., Whyte D., Hoang M.-C., Le N., Natvig J., Wingfield C., Onyeama C., Howrylak J., Toby I.T. (2023). Analysis of CDR3 Sequences from T-Cell Receptor β in Acute Respiratory Distress Syndrome. Biomolecules.

[4] Naceri S., Marc D., Blot R., Flatters D., Camproux A.-C. (2023). Druggable Pockets at the RNA Interface Region of Influenza A Virus NS1 Protein Are Conserved across Sequence Variants from Distinct Subtypes. Biomolecules.

[5] Bhowmick S., Jing T., Wang W., Zhang E.Y., Zhang F., Yang Y. (2022). In Silico Protein Folding Prediction of COVID-19 Mutations and Variants. Biomolecules.

[6] Dick A., Meuser M.E., Cocklin S. (2022). Clade-Specific Alterations within the HIV-1 Capsid Protein with Implications for Nuclear Translocation. Biomolecules.

[7] Rosignoli S., Paiardini A. (2022). Boosting the Full Potential of PyMOL with Structural Biology Plugins. Biomolecules.

